# Association of life’s essential 8 with chronic cardiovascular-kidney disorder: a prospective cohort study

**DOI:** 10.1186/s12889-024-19532-4

**Published:** 2024-09-09

**Authors:** Xinghe Huang, Jie Liang, Junyu Zhang, Jiayi Fu, Sicheng Deng, Wuxiang Xie, Fanfan Zheng

**Affiliations:** 1 School of Nursing, Chinese Academy of Medical Sciences and Peking Union Medical College, No. 33 Badachu Road, Shijingshan District, Beijing, 100144 China; 2 West China School of Nursing, Sichuan University, Sichuan, 610044 China; 3 Peking University Clinical Research Institute, Peking University First Hospital, No. 38 Xueyuan Road, Haidian District, Beijing, 100191 China; 4 Key Laboratory of Epidemiology of Major Diseases (Peking University), Ministry of Education, Beijing, 100191 China

**Keywords:** Cardiovascular health, Cardiovascular disease, Chronic kidney disease, Life’s essential 8, Modifiable risk factors

## Abstract

**Background:**

The coexistence of cardiovascular disease and chronic kidney disease, termed chronic cardiovascular-kidney disorder (CCV-KD), is increasingly prevalent. However, limited studies have assessed the association between cardiovascular health (CVH), assessed by the American Heart Association’s Life’s Essential 8 (LE8), and CCV-KD.

**Methods:**

We conducted a prospective cohort study using data from UK Biobank. Participants without cardiovascular disease and chronic kidney disease at baseline and having complete data on metrics of LE8 were included (*N* = 125,986). LE8 included eight metrics, and the aggregate score was categorized as low (< 50 points), intermediate (50 to < 80 points), and high (≥ 80 points), with a higher score indicating better CVH health. Adjusted Cox proportional hazard models were conducted to explore the association of CVH with the risk of CCV-KD. The adjusted proportion of population attributable risk (PAR%) was used to calculate the population-level risk caused by low or intermediate CVH.

**Results:**

During a median follow-up of 12.5 years, 1,054 participants (0.8%) had incident CCV-KD. Participants with intermediate and high CVH had 54% (HR = 0.46, 95% CI: 0.40–0.54, *P* < 0.001) and 75% (HR = 0.25, 95% CI: 0.18–0.34, *P* < 0.001) lower risks of incident CCV-KD compared with those in low CVH group. There was an approximately dose–response linear relationship between the overall LE8 score and incident CCV-KD. The risk of incident CCV-KD decreased by 30% (HR = 0.70, 95% CI: 0.67–0.74, *P* < 0.001) for a 10-point increment of LE8 score. The adjusted PAR% of lower overall CVH was 47.4% (95% CI: 31.6%-59.8%).

**Conclusions:**

Better CVH, assessed by using LE8 score, was strongly associated with decreased risk of incident CCV-KD. These findings imply optimizing CVH may be a preventive strategy to reduce the burden of CCV-KD.

**Supplementary Information:**

The online version contains supplementary material available at 10.1186/s12889-024-19532-4.

## Background

Cardiovascular disease (CVD) and chronic kidney disease (CKD) have become substantial public health problems globally [1, 2]. Taken individually, they are related to worsened prognosis and greater healthcare expenditures [3, 4]. It is widely recognized that CVD and CKD often coexist, and they are important risk factors for each other [5–7]. The coexistence of CVD and CKD, termed chronic cardiovascular-kidney disorder (CCV-KD), contributes additively to adverse outcomes [8, 9]. Therefore, the identification of preventive strategies is of great significance to mitigate the burden of CCV-KD as well as its morbidity and mortality.

In 2010, the concept of cardiovascular health (CVH) and the algorithm of Life’s Simple 7 (LS7) score were raised by the American Heart Association (AHA) [10]. LS7 consists of four behavioral and three biological health-related metrics, and it has been used to measure ideal CVH [10]. In 2022, the Life’s Essential 8 (LE8) was recommended by the AHA, which incorporates sleep health [11]. It employs a continuous scale with a range of 0 to 100 points for each metric, which makes it more sensitive to change over time as well as interindividual variances [12]. The relationships between CVH, assessed by LS7 or LE8, and various clinical events, including dementia, cancer, and death, have been extensively explored [13, 14].

Previous studies have investigated the associations of CVH with CVD or CKD [15–20]. Numerous studies have observed the significant association of LE8 with CVD [15, 16]. Inconsistent results have been observed between studies examining the association of LS7 with CKD [17, 18]. Recently, a cross-sectional study has reported a nonlinear association between LE8 and CKD; however, another cohort study has demonstrated a linear dose–response association [19, 20]. The different results between studies could be partially explained by different study population and study design. Although it is well acknowledged that cardiovascular and kidney disease share common risk factors and underlying pathophysiology [8]; to our knowledge, little is known about the relationship between CVH assessed by LE8 and CCV-KD. Understanding the impact of CVH on CCV-KD could help to generate feasible and effective prevention strategies to alleviate the burden of CCV-KD.

Hence, the present study aimed to explore the associations of overall CVH, assessed by LE8 score, and individual metrics with CCV-KD using data from the UK Biobank. The proportion of population attributable risk (PAR%) for lower CVH was calculated. We further explored the potential modification effect on the association between LE8 and CCV-KD.

## Methods

### Study design and population

The UK Biobank is a large-scale open-access database enrolling more than 0.5 million residents aged 40–69 years across the UK. Baseline socio-demographic information, physical measurements, biological samples, and other health-related data were collected between 2006 and 2010 [21]. Detailed information on study design has been reported previously [21]. The UK Biobank’s ethical approval was obtained from the North West Multi-centre Research Ethics Committee. All participants provided written informed consent.

In the present study, residents without complete information on LE8 metrics (*n* = 364,406), with CVD diagnosed at baseline (*n* = 8,182), with CKD diagnosed (*n* = 1,079) or eGFR < 60 mL/min/1.73 m^2^ (*n* = 1,691) at baseline, or without complete data on covariates (*n* = 1,067) were excluded, and 125,986 residents were included in our final analyses (Supplemental Fig. 1). The Chronic Kidney Disease Epidemiology Collaboration (CKD-EPI) equation was applied to calculate the eGFR [22].

**Fig. 1:**
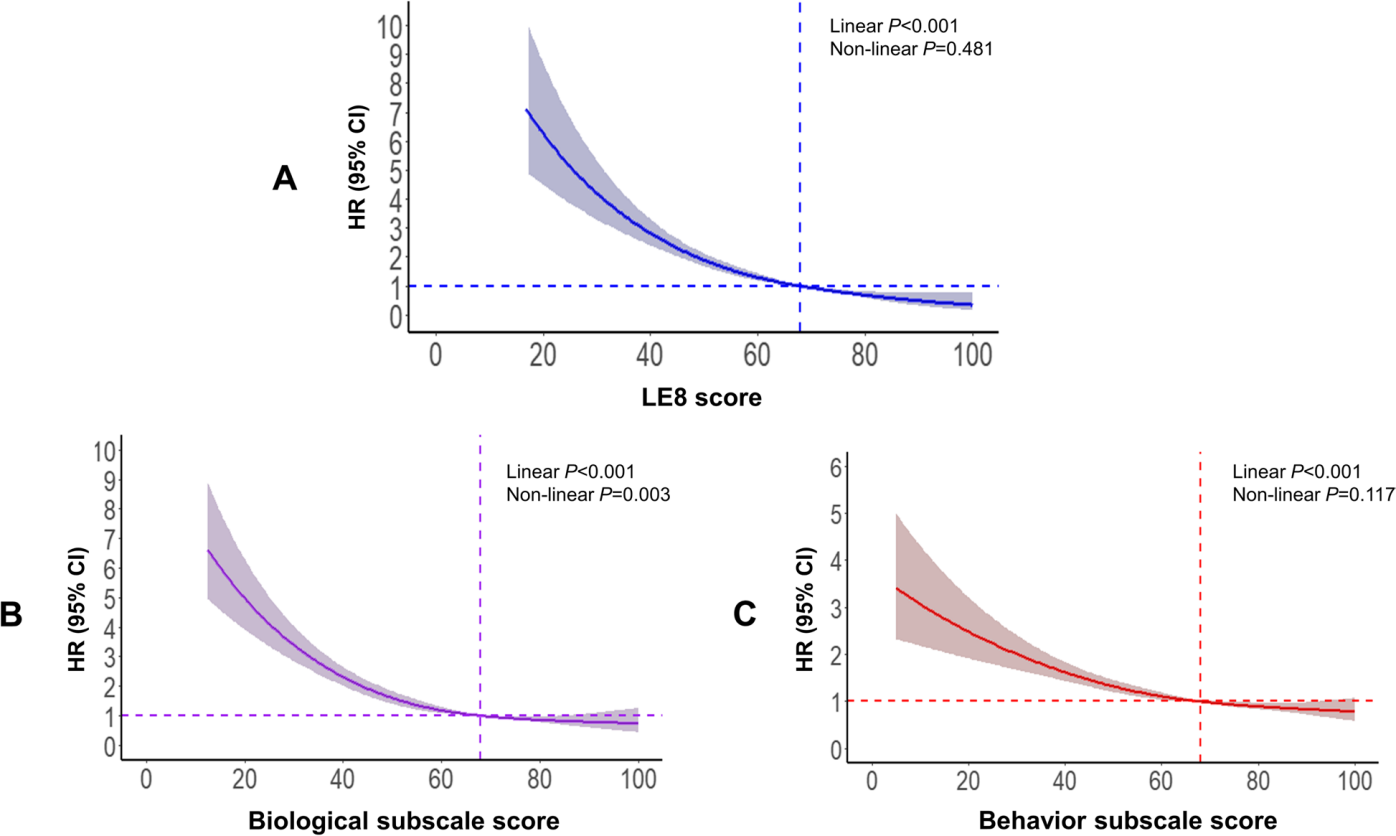
Restricted cubic spline analyses for the association of LE8 and subscales with chronic cardiovascular-kidney disorder. We used restricted cubic splines to depict the association of LE8 score (**A**), biological subscale score (**B**), and behavior subscale score (**C**) with chronic cardiovascular-kidney disorder. Multivariate adjusted models were adjusted for age, sex, ethnicity, Deprivation Index, education level, annual household income, number of morbidities, and drinking status. Solid lines indicate hazard ratios, and shaded areas indicate 95% confidence intervals. Abbreviation: CI, confidence interval; HR, hazard ratio; LE8, Life's Essential 8

### Cardiovascular health

CVH was assessed by LE8 score, including four behavior metrics (diet, physical activity, nicotine exposure, sleep health) and four biological metrics (body mass index, blood lipids, blood glucose, and blood pressure) [11]. Detailed information on the scoring methods of each cardiovascular metric is presented in Supplemental Table 1. Each metric ranges from 0 to 100 points, and the average score was calculated by adding the scores for all metrics and dividing by eight. We categorized the LE8 score as low (< 50 points), intermediate (50 to < 80 points), and high (≥ 80 points), with a higher score indicating better CVH health [11]. Individual cardiovascular metrics were also grouped as low, intermediate, and high.

**Table 1:** Baseline characteristics of participants according to cardiovascular health category

	Total	Low CVH	Intermediate CVH	High CVH	*P*‐for‐difference
N	125,986	10,577	96,337	19,072	
Age (years), mean (SD)	55.4 ± 7.9	56.1 ± 7.4	55.9 ± 7.9	52.9 ± 8.0	< 0.001
Sex					< 0.001
Female	68,105 (54.1%)	4,079 (38.6%)	50,194 (52.1%)	13,832 (72.5%)	
Male	57,881 (45.9%)	6,498 (61.4%)	46,143 (47.9%)	5,240 (27.5%)	
Ethnicity					< 0.001
White	121,347 (96.3%)	10,132 (95.8%)	92,766 (96.3%)	18,449 (96.7%)	
Others^a^	4,639 (3.7%)	445 (4.2%)	3,571 (3.7%)	623 (3.3%)	
Deprivation Index, mean (SD)	-1.6 ± 2.8	-1.1 ± 3.1	-1.7 ± 2.8	-1.9 ± 2.7	< 0.001
Education level					< 0.001
Degree or above	63,015 (50.0%)	4,016 (38.0%)	47,568 (49.4%)	11,431 (59.9%)	
Any other qualification	54,416 (43.2%)	5,347 (50.6%)	42,026 (43.6%)	7,043 (36.9%)	
No qualification	8,555 (6.8%)	1,214 (11.5%)	6,743 (7.0%)	598 (3.1%)	
Annual household income (£)					< 0.001
< 31,000	10,046 (8.0%)	766 (7.2%)	7,723 (8.0%)	1,557 (8.2%)	
≥ 31,000	42,549 (33.8%)	4,231 (40.0%)	33,280 (34.5%)	5,038 (26.4%)	
Unknown	73,391 (58.3%)	5,580 (52.8%)	55,334 (57.4%)	12,477 (65.4%)	
Multimorbidity					< 0.001
0	36,715 (29.1%)	1,857 (17.6%)	27,407 (28.4%)	7,451 (39.1%)	
1	36,253 (28.8%)	2,567 (24.3%)	28,015 (29.1%)	5,671 (29.7%)	
≥ 2	53,018 (42.1%)	6,153 (58.2%)	40,915 (42.5%)	5,950 (31.2%)	
Drinking status					< 0.001
Current	118,991 (94.4%)	9,894 (93.5%)	91,156 (94.6%)	17,941 (94.1%)	
Former	3,434 (2.7%)	433 (4.1%)	2,572 (2.7%)	429 (2.2%)	
Never	3,561 (2.8%)	250 (2.4%)	2,609 (2.7%)	702 (3.7%)	
AHA Life’s Essential 8 score, mean(SD)					
Total CVH score	67.1 ± 12.1	43.5 ± 5.4	66.2 ± 7.8	84.9 ± 4.1	< 0.001
Diet score	39.4 ± 31.1	18.1 ± 23.6	37.8 ± 30.3	59.5 ± 28.4	< 0.001
Physical activity score	80.4 ± 35.0	35.3 ± 42.2	82.1 ± 33.2	96.9 ± 12.1	< 0.001
Nicotine exposure score	63.3 ± 35.5	33.6 ± 32.5	62.0 ± 35.1	86.2 ± 22.2	< 0.001
Sleep health score	91.0 ± 16.8	81.0 ± 23.3	91.0 ± 16.5	96.1 ± 10.6	< 0.001
Body mass index score	73.2 ± 27.2	43.1 ± 27.6	72.6 ± 25.9	93.1 ± 13.7	< 0.001
Blood lipid score	49.0 ± 29.4	31.9 ± 26.0	46.2 ± 27.6	72.5 ± 27.0	< 0.001
Blood glucose score	93.4 ± 16.8	80.4 ± 25.5	93.7 ± 16.2	98.7 ± 7.6	< 0.001
Blood pressure score	47.1 ± 32.2	24.5 ± 23.0	43.8 ± 30.3	76.1 ± 26.9	< 0.001

^a^The combination of mixed, Asian, and Black participants

*Abbreviation CVH* Cardiovascular health, *AHA* American Heart Association

ANOVA and χ^2^ test were used to test the differences among categories for continuous and categorical variables, respectively

### Chronic cardiovascular-kidney disorder

The outcome of CCV-KD was defined as the coexistence of CVD (including stroke, heart failure, coronary heart disease, and atrial fibrillation) and CKD [8]. The diagnoses of these diseases were ascertained through linkage from inpatient records, self-reported data, and death registry. The variable ID of these diseases in UK Biobank can be found in Supplemental Table 2. The occurrence date of CCV-KD was determined as the diagnosis date of CVD if CKD has been diagnosed before or the diagnosis date of CKD if CVD has been diagnosed before. Follow-up time was calculated from the enrollment date to the date of diagnosis of CCV-KD, or death, or the end of follow-up (December 31, 2021), whichever occurred first.

### Covariates

Study covariates included age, sex (female, male), ethnicity (white, the combination of mixed, Asian, and Black participants), Townsend Deprivation Index, education level (degree or above, other qualification, no qualification), annual household income (less than £31,000, greater than or equal to £31,000, unknown), number of morbidities (0, 1, ≥ 2), and drinking status (current, former, never). The definition and assessment method of covariates can be found in Supplemental Table 2.

### Statistical analysis

Participants’ characteristics were reported as mean (standard deviation) or frequency (percentage). Comparisons of differences between CVH categories (low, intermediate, and high) were made with ANOVA or χ^2^ test as appropriate.

The cumulative incidence of CCV-KD was calculated by the Kaplan–Meier method using the log-rank test. We used Cox proportional hazard models to examine the association of CVH category with CCV-KD. In model 1, we adjusted for age and sex. In model 2, we additionally adjusted for ethnicity, Townsend Deprivation Index, education level, and annual household income. In model 3, we further added the number of morbidities and drinking status. Additionally, we performed Cox regression analyses to determine the association of behavior subscale, biological subscale, and 8 individual metrics with incident CCV-KD. The adjusted PAR% of high (≥ 80 points) versus intermediate or low CVH (< 80 points) was estimated for the proportion of CCV-KD that would be avoided if all participants were in high CVH category [23]. We also estimated PAR% for individual cardiovascular metrics. We then examined the association between 10-point increments of LE8 score and incident CCV-KD. Additionally, restricted cubic splines were applied to assess the associations of continuous LE8 score, behavior and biological subscale scores, with CCV-KD with 4 knots using the median score of 68 points as the reference.

Subgroup analyses were conducted to explore the association stratified by sex, age, ethnicity, deprivation, education level, annual household income, number of morbidities, and drinking status. Several sensitivity analyses were also performed. First, we grouped participants according to the quartiles of LE8 score. Second, we conducted Fine-Gray analyses in consideration of death as the competing risk on the association between overall CVH and incident CCV-KD. Third, participants who developed CCV-KD within two years after baseline were excluded to avoid potential reverse causality. Fourth, we applied multiple imputations to handle missing values on covariates.

A 2-tailed *P* < 0.05 was deemed statistically significant. All analyses were conducted by SAS 9.4 and R software, version 4.2.2.

## Results

### Participants characteristics

A total of 125,986 participants (mean age 55.4 ± 7.9 years, 54.1% female) were included in this study (Supplemental Fig. 1). Participants’ characteristics according to CVH group are presented in Table 1. Overall, 8.4%, 76.5%, and 15.1% had low, intermediate, and high CVH, respectively. Adults having higher CVH were younger, having a higher proportion of females, higher education levels, and fewer morbidities (Table 1).

### Cardiovascular health and incident chronic cardiovascular-kidney disorder

Incident CCV-KD occurred in 1,054 participants (0.8%) over a median follow-up of 12.5 years (IQR: 11.9–13.2 years). The cumulative incidence of incident CCV-KD was lowest in the high CVH group (log-rank *P* < 0.001) compared with lower CVH groups (Supplemental Fig. 2). Participants having intermediate and high CVH had 54% (HR = 0.46, 95% CI: 0.40–0.54, *P* < 0.001) and 75% (HR = 0.25, 95% CI: 0.18–0.34, *P* < 0.001) lower risk of incident CCV-KD compared with those having low CVH (Supplemental Table 3). Similar associations were observed between subscales and incident CCV-KD (Supplemental Table 4). A 10-point increase in LE8 score resulted in a 30% reduction in the risk of incident CCV-KD (HR = 0.70, 95% CI: 0.67–0.74, *P* < 0.001) (Supplemental Table 5). The multivariable adjusted restricted cubic spline confirmed a linear association of LE8 score with incident CCV-KD (*P*_nonlinear_ = 0.481, *P*_linear_ < 0.001) (Fig. 1). The risk of CCV-KD was higher among participants with lower biological subscale score or behavior subscale score (Fig. 1).

**Fig. 2:**
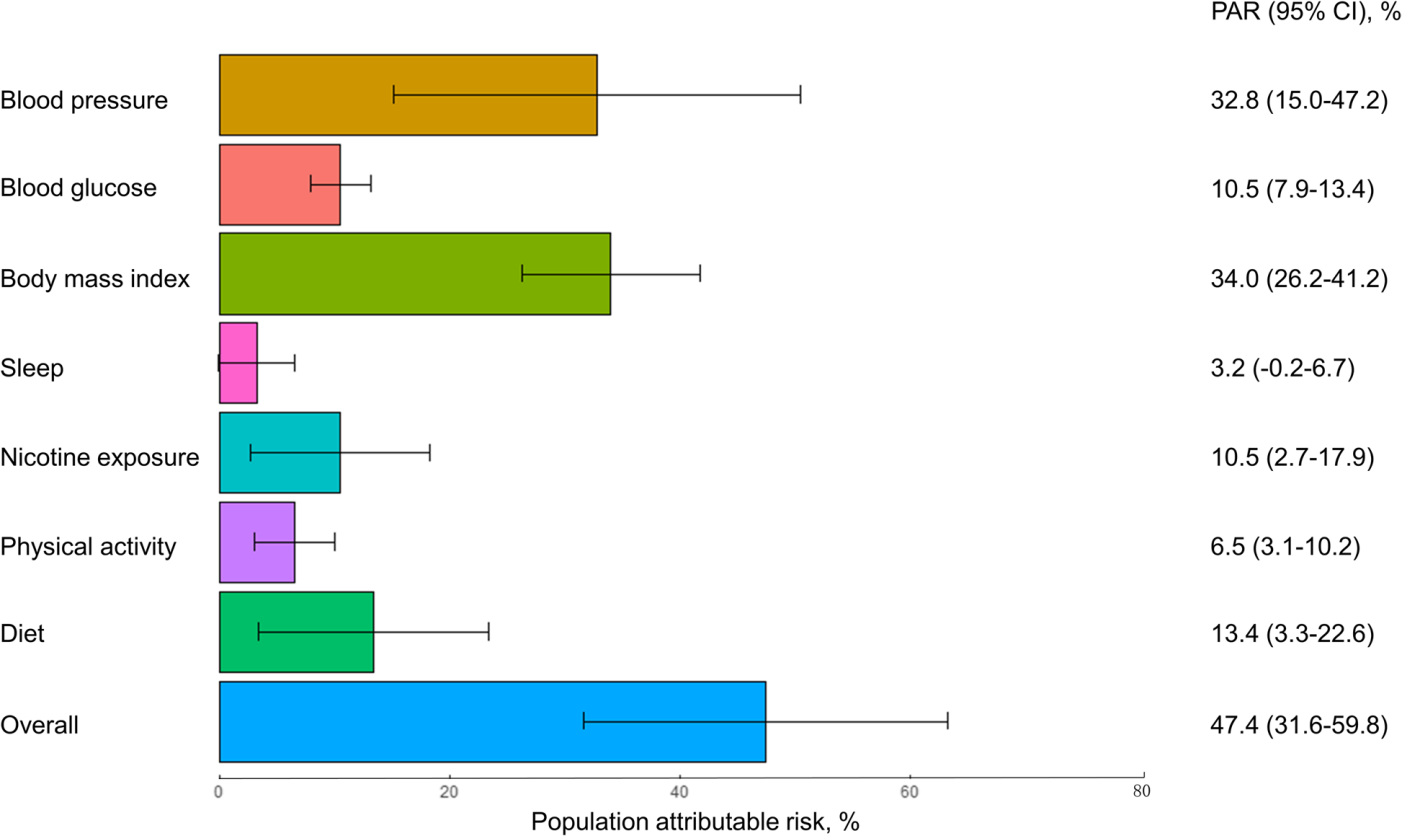
The proportion of population attributable risk of each individual metric of LE8. Models were adjusted for age, sex, ethnicity, Deprivation Index, education level, annual household income, number of morbidities, and drinking status. Abbreviation:; LE8, Life's Essential 8; PAR, population attributable risk

A decreased risk of CCV-KD was also observed among participants with higher individual metric scores, except for the blood lipid score (Supplemental Table 3). The adjusted PAR% related to low or intermediate overall CVH was 47.4% (95% CI: 31.6%-59.8%). Regarding individual CVH metrics, body mass index showed the highest PAR% (34.0%, 95% CI: 26.2%-41.2%) (Fig. 2).

### Subgroup and sensitivity analyses

**Fig. 3:**
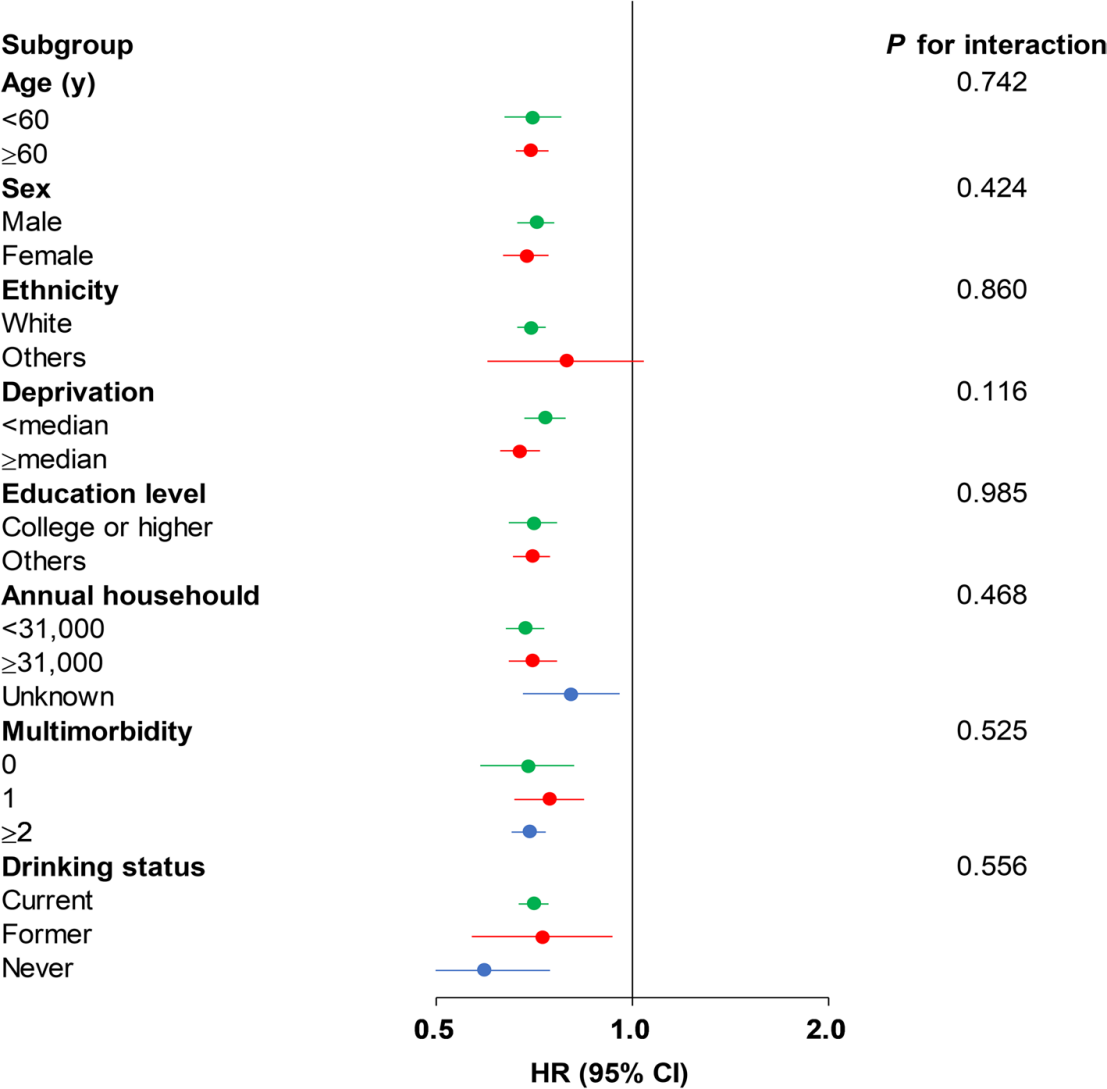
Subgroup analysis of the association between 10-point increase of LE8 score and chronic cardiovascular-kidney disorder Forest plots displaying hazard ratios and 95% confidence intervals for chronic cardiovascular-kidney disorder with 10-point increase of LE8 score. Abbreviation: CI: confidence interval; HR: hazard ratio; LE8, Life’s Essential 8

We did not find the modification effect of sex, age, ethnicity, and other covariates on the association of CVH with CCV-KD (*P* > 0.05 for interaction) (Fig. 3 and Supplemental Table 6). After dividing participants into four groups based on LE8 quartiles, the relationship between higher LE8 score quartiles and decreased CCV-KD risk has been observed (Supplemental Table 5). Similar results were observed when taking competing risk of death into consideration (Supplemental Table 7). After excluding incident CCV-KD cases within the initial two years of follow-up (*n* = 11), the association was not substantially altered (Supplemental Table 8). Additionally, the results remain consistent after multiple imputation analyses for covariates with missing data (Supplemental Table 9).

Among 1,054 participants with CCV-KD during the follow-up period, 565 participants were diagnosed with CVD first, 297 participants were diagnosed with CKD first, and 192 participants were diagnosed with CVD and CKD at the same date. Regardless of the disease diagnosed first, the association between CVH and CCV-KD was statistically significant (Supplemental Table 10).

## Discussion

Among 125,986 participants without CVD and CKD at baseline, we found an association between CVH, assessed using LE8 score, and individual health metrics with incident CCV-KD. Participants with intermediate and high CVH had 54% and 75% lower risk, respectively, of incident CCV-KD compared with those having low CVH. We observed a dose–response linear association between LE8 score and incident CCV-KD. The adjusted PAR% revealed 47.4% of CCV-KD could be avoided if all participants could achieve high LE8 score. Consistent findings have been found in subgroups, including age, sex, and ethnicity. These findings suggest optimizing CVH could be a feasible preventive strategy for incident CCV-KD, emphasizing the importance of applying a simple algorithm such as LE8 to evaluate CVH level and taking effective interventions to promote CVH.

To the best of our knowledge, the current study is the first demonstration of the association of CVH assessed by LE8 score with CCV-KD among the general population. The concept of cardiorenal syndrome has been widely adopted before [24]. Recently, a new term of CCV-KD has been put forward based on the common risk factors and shared pathophysiological mechanisms of CVD and CKD [8]. In 2022, the concept of LE8, including four behavior metrics and four biological metrics, was proposed by the AHA, and it provides a more holistic and detailed evaluation of CVH at individual and population levels than LS7 [11]. Several studies have reported that higher LE8 score was related to reduced risk of incident CVD [15, 16]. Based on the UK Biobank, a previous study found participants with high CVH had 64% lower risk of CVD compared with the low CVH group [15]. In the Kailuan cohort study, LE8 was related to premature CVD among Chinese adults [16]. Conflicting findings have been observed in the association between LE8 and CKD risk. Ren et al. reported a negative and nonlinear association between LE8, as well as its subscales, and the prevalence of CKD [19]; however, Tang et al. found a linear association of overall LE8 score and subscales with the risk of CKD [20]. The differences could be partly explained by study population, study design, and sample size. This large-scale cohort study extends existing literature as it suggested a significant linear association of LE8 score with the risk of CCV-KD, and almost half of the cases could be prevented if they can obtain high LE8 score. These findings indicated that maintaining better CVH is of great importance to prevent subsequent CCV-KD among the general population.

With regard to the individual health metrics, we demonstrated that body mass index was the leading individual factor for CCV-KD. Numerous studies have shown that elevated adiposity indicators were independently related to cardiovascular risk and eGFR decline [25, 26]. Consistent with previous findings, our results indicated that lower body mass index was related to decreased risk of CCV-KD [27]. Some large-scale research has suggested the link between obesity and CVD or CKD is mediated by diabetes, hypertension, or other comorbidities [28, 29]; however, other studies showed significant residual risks of obesity [30]. Indeed, diabetes and hypertension are the most common risk factors for CVD and CKD [31, 32]. There is a number of studies showing that improving blood pressure and glycemic control may improve vascular and renal outcomes [31, 32]. Our study also identified the significant protective effect of lower blood pressure and blood glucose. The possible mechanisms by which these biological metrics may impact the development of CCV-KD could be intertwined, underlining the significance of the holistic management of these risk factors.

Interestingly, there was a U-shaped association between non-HDL cholesterol score in LE8 and CCV-KD risk, with both low and high blood lipid scores having high risk. The CRIC cohort study among the US population reported blood lipid score in LS7 was not associated with CKD risk [18], while other studies have shown a U-shaped curve of risk where the risk of CKD increased among participants with low and high non-HDL cholesterol levels [33, 34]. Inconsistent results have also been found in the association of blood lipids with the risk of cardiovascular events. A prospective cohort study found high blood lipid score in LE8 was associated with a lower risk of CVD [35]; however, another study suggested a U-shaped relationship between non-HDL cholesterol levels and CVD death [36]. The underlying mechanism is unclear and a possible explanation could be that elevated HDL cholesterol may paradoxically accelerate impaired endothelial progenitor cell tube formation and angiogenesis, leading to inflammation and oxidative stress, which could lead to microvascular disease and renal dysfunction [37, 38]. Future research is warranted to further examine the underpinning mechanism of the association.

Our study also found health behaviors were important contributors to incident CCV-KD. Physical activity is a critical aspect of lifestyle modification which has a beneficial effect on CVD and CKD progression [39, 40]. Our data validated the association of physical activity score as an individual health metric in LE8 with CCV-KD and underscored its value on lowering the risk of CCV-KD. In the new algorithm of CVH (LE8), secondhand smoke exposure has been incorporated into the nicotine exposure metric [11]. In line with other studies, our study showed a detrimental impact of smoking on CCV-KD among the general population, indicating smoking cessation also plays a significant part in the primary prevention of CCV-KD [41, 42]. In addition, we found the beneficial impact of a healthy diet in LE8 on the reduction of CCV-KD risk; however, the CRIC cohort study did not observe the significant association of a healthy diet pattern in LS7 with incident CKD [18]. The inconsistency between studies could be partly ascribed to different study populations and different methods of assessing dietary quality.

In recent years, there has been an increasing number of research focusing on sleep health, and sleep duration has been added as the eighth metric to define CVH in LE8 [11]. Our study indicated that increased sleep health score was associated with decreased risk of CCV-KD, which was compatible with previous studies detecting the association between healthy sleep pattern and reduced CVD or CKD risk [43–45]. However, in our study, the adjusted PAR% was smaller than other health metrics (< 5%). Similarly, Tang et al. and Sun et al. found the adjusted PAR% related to sleep health with CKD and all-cause death were small (3.2% and 5.4%) [20, 46]. Therefore, research on the public health importance of sleep health to clinical outcomes, especially for death, CVD, and CKD, warrants further investigation.

### Strengths and limitations

The current study had several strengths. This is the first study to explore the association of CVH, assessed by LE8 score, and individual health metrics with incident CCV-KD. We also firstly estimated the adjusted PAR% of overall CVH and individual metrics with CCV-KD. Additionally, the robustness of the results was demonstrated by the consistent results between the main analyses and several sensitivity analyses. Nevertheless, these findings need to be interpreted in light of some limitations. First, the nature of observational research makes it difficult to determine the causal relationship between CVH and CCV-KD even if consistent results were observed after excluding individuals developing CCV-KD within the initial two years of follow-up. Second, lifestyle factors, such as physical activity and dietary habits, were evaluated based on self-reported information, which may introduce recall and misclassification bias. Third, we did not explore the association between changes of CVH over time and incident CCV-KD given that most CVH information was only gathered at baseline, but prior research indicated CVH levels are mainly stable or decline over time [47]. The association could be biased toward the null owing to the possibility of misclassification over time. Fourth, this study involved residents who were mostly of European descent, limiting the generalizability of our findings to other ethnicities.

## Conclusions

Higher CVH, assessed by LE8 score, is significantly associated with lower risk of incident CCV-KD. Body mass index, blood pressure, and diet were the most important health metrics for incident CCV-KD. Our findings reveal the potential clinical benefits of optimizing LE8 for CCV-KD prevention. Further efforts should be made to identify effective strategies to promote CVH metrics.

## Supplementary Information

Supplementary Material 1.

## Data Availability

The data used for analysis in this study is available from UK Biobank project site, subject to registration and application process. Further details can be found at https://www.ukbiobank.ac.uk.

## Abbreviations

AHA: American Heart Association
CCV-KD: Chronic cardiovascular-kidney disorder
CKD: Chronic kidney disease
CKD-EPI: Chronic Kidney Disease Epidemiology Collaboration
CVD: Cardiovascular disease
CVH: Cardiovascular health
eGFR: Estimated glomerular filtration rate
HDL: High-density lipoprotein
LE8: Life’s Essential 8
LS7: Life’s Simple 7
PAR: Population attributable risk
UK: United Kingdom

## References

[1] GBD Chronic Kidney Disease Collaboration. Global, regional, and national burden of chronic kidney disease, 1990–2017: a systematic analysis for the Global Burden of Disease Study 2017. The Lancet. 2020;395(10225):709–33. 10.1016/S0140-6736(20)30045-3. 10.1016/S0140-6736(20)30045-3 PMC7049905 32061315

[2] Foreman KJ, Marquez N, Dolgert A, et al. Forecasting life expectancy, years of life lost, and all-cause and cause-specific mortality for 250 causes of death: reference and alternative scenarios for 2016–40 for 195 countries and territories. The Lancet. 2018;392(10159):2052–90. 10.1016/S0140-6736(18)31694-5. 10.1016/S0140-6736(18)31694-5 PMC6227505 30340847

[3] Tsao CW, Aday AW, Almarzooq ZI. American Heart Association Council on Epidemiology and Prevention Statistics Committee and Stroke Statistics Subcommittee. Heart disease and stroke statistics-2023 update: A report from the American Heart Association. *Circulation*. 2023;147(8):e93-e621. 10.1161/CIR.0000000000001123. 10.1161/CIR.0000000000001123 PMC12135016 36695182

[4] Muka T, Imo D, Jaspers L, et al. The global impact of non-communicable diseases on healthcare spending and national income: a systematic review. Eur J Epidemiol. 2015;30(4):251–77. 10.1007/s10654-014-9984-2. 25595318 10.1007/s10654-014-9984-2

[5] Marassi M, Fadini GP. The cardio-renal-metabolic connection: a review of the evidence. Cardiovasc Diabetol. 2023;22(1):195. 10.1186/s12933-023-01937-x. 37525273 10.1186/s12933-023-01937-x PMC10391899

[6] Zoccali C, Mallamaci F, Adamczak M, et al. Cardiovascular complications in chronic kidney disease: a review from the European Renal and Cardiovascular Medicine Working Group of the European Renal Association. Cardiovasc Res. 2023;119(11):2017–32. 10.1093/cvr/cvad083. 37249051 10.1093/cvr/cvad083 PMC10478756

[7] Al-Wahsh H, Tangri N, Quinn R, et al. Accounting for the competing risk of death to predict kidney failure in adults with stage 4 chronic kidney disease. JAMA Netw Open. 2021;4(5): e219225. 10.1001/jamanetworkopen.2021.9225. 33944922 10.1001/jamanetworkopen.2021.9225 PMC8097501

[8] Zoccali C, Mallamaci F, Halimi JM, et al. Chronic cardiovascular-kidney disorder: a new conceptual framework. *Nat Rev Nephrol*. 2023 Nov 15. Epub ahead of print. 10.1038/s41581-023-00789-8. 10.1038/s41581-023-00789-8 37968510

[9] Halimi JM, de Fréminville JB, Gatault P, Bisson A, et al. Long-term impact of cardiorenal syndromes on major outcomes based on their chronology: a comprehensive French nationwide cohort study. Nephrol Dial Transplant. 2022;37(12):2386–97. 10.1093/ndt/gfac153. 35438794 10.1093/ndt/gfac153

[10] Lloyd-Jones DM, Hong Y, Labarthe D, et al. Defining and setting national goals for cardiovascular health promotion and disease reduction: the American Heart Association’s strategic Impact Goal through 2020 and beyond. Circulation. 2010;121(4):586–613. 10.1161/CIRCULATIONAHA.109.192703. 20089546 10.1161/CIRCULATIONAHA.109.192703

[11] Lloyd-Jones DM, Allen NB, Anderson CAM, et al; American Heart Association. Life’s Essential 8: Updating and enhancing the American Heart Association’s Construct of Cardiovascular Health: a presidential advisory from the American Heart Association. *Circulation*. 2022;146(11):e18-e43. 10.1161/CIR.0000000000001078. 10.1161/CIR.0000000000001078 PMC10503546 35766027

[12] Lloyd-Jones DM, Ning H, Labarthe D, et al. Status of cardiovascular health in US adults and children using the American Heart Association’s New “Life’s Essential 8” Metrics: prevalence estimates from the National Health and Nutrition Examination Survey (NHANES), 2013 through 2018. Circulation. 2022;146(11):822–35. 10.1161/CIRCULATIONAHA.122.060911. 35766033 10.1161/CIRCULATIONAHA.122.060911

[13] Wang X, Ma H, Li X, Heianza Y, et al. Association of cardiovascular health with life expectancy free of cardiovascular disease, diabetes, cancer, and dementia in UK adults. JAMA Intern Med. 2023;183(4):340. 10.1001/jamainternmed.2023.0015. 36848126 10.1001/jamainternmed.2023.0015 PMC9972243

[14] Yang Q, Cogswell ME, Flanders WD, et al. Trends in cardiovascular health metrics and associations with all-cause and CVD mortality among US adults. JAMA. 2012;307(12):1273. 10.1001/jama.2012.339. 22427615 10.1001/jama.2012.339 PMC9004324

[15] Li X, Ma H, Wang X, Feng H, Qi L. Life’s Essential 8, genetic susceptibility, and incident cardiovascular disease: A prospective study. Arterioscler Thromb Vasc Biol. 2023;43(7):1324–33. 10.1161/ATVBAHA.123.319290. 37199161 10.1161/ATVBAHA.123.319290 PMC10330462

[16] Rempakos A, Prescott B, Mitchell GF, Vasan RS, Xanthakis V. Association of Life’s Essential 8 with cardiovascular disease and mortality: The Framingham Heart Study. J Am Heart Assoc. 2023;12(23): e030764. 10.1161/JAHA.123.030764. 38014669 10.1161/JAHA.123.030764 PMC10727315

[17] Muntner P, Judd SE, Gao L, et al. Cardiovascular risk factors in CKD associate with both ESRD and mortality. J Am Soc Nephrol. 2013;24(7):1159–65. 10.1681/ASN.2012070642. 23704285 10.1681/ASN.2012070642 PMC3699822

[18] Ricardo AC, Anderson CA, Yang W, et al. Healthy lifestyle and risk of kidney disease progression, atherosclerotic events, and death in CKD: findings from the Chronic Renal Insufficiency Cohort (CRIC) Study. Am J Kidney Dis. 2015;65(3):412–24. 10.1053/j.ajkd.2014.09.016. 25458663 10.1053/j.ajkd.2014.09.016 PMC4339665

[19] Ren Y, Cai Z, Guo C, et al. Associations between Life’s Essential 8 and chronic kidney disease. J Am Heart Assoc. 2023;12(24): e030564. 10.1161/JAHA.123.030564. 38063194 10.1161/JAHA.123.030564 PMC10863789

[20] Tang R, Wang X, Li X, et al. Adherence to Life’s Essential 8 and incident chronic kidney disease: a prospective study of 147,988 UK Biobank participants. Am J Clin Nutr. 2023;118(4):804–11. 10.1016/j.ajcnut.2023.08.007. 37604298 10.1016/j.ajcnut.2023.08.007 PMC10579043

[21] Palmer LJ. UK Biobank: bank on it. The Lancet. 2007;369(9578):1980–2. 10.1016/S0140-6736(07)60924-6. 10.1016/S0140-6736(07)60924-6 17574079

[22] Levey AS, Stevens LA, Schmid CH, et al; CKD-EPI (Chronic Kidney Disease Epidemiology Collaboration). A new equation to estimate glomerular filtration rate. Ann Intern Med. 2009;150(9):604. 10.7326/0003-4819-150-9-200905050-00006. 10.7326/0003-4819-150-9-200905050-00006 PMC2763564 19414839

[23] Spiegelman D, Hertzmark E, Wand HC. Point and interval estimates of partial population attributable risks in cohort studies: examples and software. Cancer Causes Control. 2007;18(5):571–9. 10.1007/s10552-006-0090-y. 17387622 10.1007/s10552-006-0090-y

[24] Ronco C, Haapio M, House AA, Anavekar N, Bellomo R. Cardiorenal syndrome. J Am Coll Cardiol. 2008;52(19):1527–39. 10.1016/j.jacc.2008.07.051. 19007588 10.1016/j.jacc.2008.07.051

[25] Powell-Wiley TM, Poirier P, Burke LE, et al. Obesity and cardiovascular disease: A scientific statement from the American Heart Association. Circulation. 2021;143(21):e984–1010. 10.1161/CIR.0000000000000973. 33882682 10.1161/CIR.0000000000000973 PMC8493650

[26] Chang AR, Grams ME, Ballew SH, et al. Adiposity and risk of decline in glomerular filtration rate: meta-analysis of individual participant data in a global consortium. BMJ. 2019;364: k5301. 10.1136/bmj.k5301. 30630856 10.1136/bmj.k5301 PMC6481269

[27] Herrington WG, Smith M, Bankhead C, et al. Body-mass index and risk of advanced chronic kidney disease: Prospective analyses from a primary care cohort of 1.4 million adults in England. PloS One. 2017;12(3):e0173515. 10.1371/journal.pone.0173515. 10.1371/journal.pone.0173515 PMC5342319 28273171

[28] Ndumele CE, Matsushita K, Lazo M, et al. Obesity and subtypes of incident cardiovascular disease. J Am Heart Assoc. 2016;5(8): e003921. 10.1161/JAHA.116.003921. 27468925 10.1161/JAHA.116.003921 PMC5015307

[29] Hubert HB, Feinleib M, McNamara PM, Castelli WP. Obesity as an independent risk factor for cardiovascular disease: a 26-year follow-up of participants in the Framingham Heart Study. Circulation. 1983;67(5):968–77. 10.1161/01.cir.67.5.968. 6219830 10.1161/01.cir.67.5.968

[30] Wilson PW, Bozeman SR, Burton TM, Hoaglin DC, Ben-Joseph R, Pashos CL. Prediction of first events of coronary heart disease and stroke with consideration of adiposity. Circulation. 2008;118(2):124–30. 10.1161/CIRCULATIONAHA.108.772962. 18591432 10.1161/CIRCULATIONAHA.108.772962

[31] Webster AC, Nagler EV, Morton RL, Masson P. Chronic kidney disease. The Lancet. 2017;389(10075):1238–52. 10.1016/S0140-6736(16)32064-5. 10.1016/S0140-6736(16)32064-5 27887750

[32] Yen FS, Wei JC, Chiu LT, Hsu CC, Hwu CM. Diabetes, hypertension, and cardiovascular disease development. J Transl Med. 2022;20(1):9. 10.1186/s12967-021-03217-2. 34980154 10.1186/s12967-021-03217-2 PMC8722333

[33] Suh SH, Oh TR, Choi HS, et al; Korean Cohort Study for Outcomes in Patients with Chronic Kidney Disease (KNOW-CKD) Investigators. Non-high-density lipoprotein cholesterol and progression of chronic kidney disease: results from the KNOW-CKD Study. Nutrients. 2022;14(21):4704. 10.3390/nu14214704.

[34] Kintu C, Soremekun O, Kamiza AB, et al. The causal effects of lipid traits on kidney function in Africans: bidirectional and multivariable Mendelian-randomization study. eBioMedicine. 2023;90:104537. 10.1016/j.ebiom.2023.104537. 10.1016/j.ebiom.2023.104537 PMC10070509 37001235

[35] Xing A, Tian X, Wang Y, et al. ‘Life’s Essential 8’ cardiovascular health with premature cardiovascular disease and all-cause mortality in young adults: the Kailuan prospective cohort study. Eur J Prev Cardiol. 2023;30(7):593–600. 10.1093/eurjpc/zwad033. 36757396 10.1093/eurjpc/zwad033

[36] Zeng RX, Xu JP, Kong YJ, Tan JW, Guo LH, Zhang MZ. U-shaped relationship of non-HDL cholesterol with all-cause and cardiovascular mortality in men without statin therapy. Front Cardiovasc Med. 2022;9: 903481. 10.3389/fcvm.2022.903481. 35872887 10.3389/fcvm.2022.903481 PMC9300868

[37] Huang CY, Lin FY, Shih CM, et al. Moderate to high concentrations of high-density lipoprotein from healthy subjects paradoxically impair human endothelial progenitor cells and related angiogenesis by activating Rho-associated kinase pathways. Arterioscler Thromb Vasc Biol. 2012;32(10):2405–17. 10.1161/ATVBAHA.112.248617. 22904272 10.1161/ATVBAHA.112.248617

[38] Bowe B, Xie Y, Xian H, Balasubramanian S, Al-Aly Z. Low levels of high-density lipoprotein cholesterol increase the risk of incident kidney disease and its progression. Kidney Int. 2016;89(4):886–96. 10.1016/j.kint.2015.12.034. 26924057 10.1016/j.kint.2015.12.034

[39] Dempsey PC, Rowlands AV, Strain T, et al. Physical activity volume, intensity, and incident cardiovascular disease. Eur Heart J. 2022;43(46):4789–800. 10.1093/eurheartj/ehac613. 36302445 10.1093/eurheartj/ehac613

[40] Kalantar-Zadeh K, Jafar TH, Nitsch D, Neuen BL, Perkovic V. Chronic kidney disease. The Lancet. 2021;398(10302):786–802. 10.1016/S0140-6736(21)00519-5. 10.1016/S0140-6736(21)00519-5 34175022

[41] Jeong SM, Jeon KH, Shin DW, et al. Smoking cessation, but not reduction, reduces cardiovascular disease incidence. Eur Heart J. 2021;42(40):4141–53. 10.1093/eurheartj/ehab578. 34431997 10.1093/eurheartj/ehab578

[42] Xia J, Wang L, Ma Z, et al. Cigarette smoking and chronic kidney disease in the general population: a systematic review and meta-analysis of prospective cohort studies. Nephrol Dial Transplant. 2017;32(3):475–87. 10.1093/ndt/gfw452. 28339863 10.1093/ndt/gfw452

[43] Geng T, Li X, Ma H, Heianza Y, Qi L. Adherence to a healthy sleep pattern and risk of chronic kidney disease: the UK Biobank Study. Mayo Clin Proc. 2022;97(1):68–77. 10.1016/j.mayocp.2021.08.028. 34996567 10.1016/j.mayocp.2021.08.028 PMC8851869

[44] Zhang H, Wang B, Chen C, et al. Sleep patterns, genetic susceptibility, and incident chronic kidney disease: a prospective study of 370 671 participants. Front Neurosci. 2022;16: 725478. 10.3389/fnins.2022.725478. 35173575 10.3389/fnins.2022.725478 PMC8843034

[45] Fan M, Sun D, Zhou T, et al. Sleep patterns, genetic susceptibility, and incident cardiovascular disease: a prospective study of 385 292 UK biobank participants. Eur Heart J. 2020;41(11):1182–9. 10.1093/eurheartj/ehz849. 31848595 10.1093/eurheartj/ehz849 PMC7071844

[46] Sun J, Li Y, Zhao M, et al. Association of the American Heart Association’s new “Life’s Essential 8” with all-cause and cardiovascular disease-specific mortality: prospective cohort study. BMC Med. 2023;21(1):116. 10.1186/s12916-023-02824-8. 36978123 10.1186/s12916-023-02824-8 PMC10053736

[47] Enserro DM, Vasan RS, Xanthakis V. Twenty-year trends in the American Heart Association cardiovascular health score and impact on subclinical and clinical cardiovascular disease: the Framingham Offspring Study. J Am Heart Assoc. 2018;7(11): e008741. 10.1161/JAHA.118.008741. 29773573 10.1161/JAHA.118.008741 PMC6015351

