# Supplementary material for: Association of life’s essential 8 with chronic cardiovascular-kidney disorder: a prospective cohort study
Source: BMC Public Health. 2024 Sep 9;24:2448. doi: 10.1186/s12889-024-19532-4 (PMC11382523; doi:10.1186/s12889-024-19532-4)

**Supplemental Material**

**Association of Life’s Essential 8 with chronic cardiovascular-kidney disorder: a prospective cohort study**

[Supplemental Table 1 The scoring method of eight health metrics of Life’s Essential 8 score 1](#_Toc170912860)

[Supplemental Table 2 Definition and assessment of variables 3](#_Toc170912861)

[Supplemental Table 3 The risk of chronic cardiovascular-kidney disorder according to cardiovascular health score 5](#_Toc170912862)

[Supplemental Table 4 Association between CVH subscales and risk of CCV-KD 7](#_Toc170912863)

[Supplemental Table 5 Association between CVH quartiles and risk of CCV-KD 8](#_Toc170912864)

[Supplemental Table 6 Association between CVH categories and risk of CCV-KD in subgroups 9](#_Toc170912865)

[Supplemental Table 7 Association between CVH categories and risk of CCV-KD using competing risk models 11](#_Toc170912866)

[Supplemental Table 8 Association between CVH categories and risk of CCV-KD excluding events within the first two years of follow-up 12](#_Toc170912867)

[Supplemental Table 9 Association between CVH categories and risk of CCV-KD after imputing missing values on covariates 13](#_Toc170912868)

[Supplemental Table 10 The risk of chronic cardiovascular-kidney disorder separated by the disease diagnosed first according to CVH score 14](#_Toc170912869)

[Supplemental Figure 1 Flowchart of the study 16](#_Toc170912870)

[Supplemental Figure 2 Kaplan-Meier curve for cumulative incidence of chronic cardiovascular-kidney disorder by cardiovascular health category 17](#_Toc170912871)

## Supplemental Table 1 The scoring method of eight health metrics of Life’s Essential 8 score

| **Metric** | **Method of measurement** | **Quantification** | |
| --- | --- | --- | --- |
| **Diet** **score** | Self-reported daily intake of a DASH-style eating pattern.  Using dietary data collected from 24h dietary recalls conducted using the Oxford WebQ between 2009 and 2012. | 100 | 95th percentile |
|  |  | 80 | 75th-94th percentile |
|  |  | 50 | 50th-74th percentile |
|  |  | 25 | 25th-49th percentile |
|  |  | 0 | 1st-24th percentile |
| **Physical activity score** | Self-reported physical activity converted to MET/week. | 100 | ≥600 MET/min/week |
|  |  | 90 | ≥480 to <600 MET/min/week |
|  |  | 80 | ≥360 to <480 MET/min/week |
|  |  | 60 | ≥240 to <360 MET/min/week |
|  |  | 40 | ≥120 to <240 MET/min/week |
|  |  | 20 | ≥4 to <120 MET/min/week |
|  |  | 0 | 0 MET/min/week |
| **Nicotine exposure** **score** | Self-reported use of cigarettes (current smoking status and history of smoking) or secondhand smoke exposure. | 100 | Never smoker |
|  |  | 75 | Former smoker, quit ≥5 years |
|  |  | 50 | Former smoker, quit 1-<5 years |
|  |  | 25 | Former smoker, quit <1years |
|  |  | 0 | Current smoker |
|  |  | Subtract 20 points (unless score is 0) for living with active indoor smoker in home. In addition, the information on the specific time to quit smoking was only available among participants who indicated “smoked on most or all days in the past”. We considered the participants who indicated “smoked occasionally in the past” as equivalent to “former smoker, quit 1-<5 years”; We considered the participants who indicated “ just tried once or twice in the past” as equivalent to “former smoker, quit ≥ 5 years”. | |
|  |  |  |  |
|  |  |  |  |
|  |  |  |  |
|  |  |  |  |
|  |  |  |  |
|  |  |  |  |
| **Sleep health score** | Self-reported average hours of sleep per night. | 100 | 7-9 hours |
|  |  | 90 | 9-<10 hours |
|  |  | 70 | 6-<7 hours |
|  |  | 40 | 5-<6 or ≥10 hours |
|  |  | 20 | 4-<5 hours |
|  |  | 0 | < 4 hours |
| **Body mass index score** | Body mass index: body weight (kilograms) divided by height squared (meters squared). | 100 | <25 kg/m^2^ |
|  |  | 70 | 25.0-29.9 kg/m^2^ |
|  |  | 30 | 30.0-34.9 kg/m^2^ |
|  |  | 15 | 35.0-39.9 kg/m^2^ |
|  |  | 0 | ≥ 40.0 kg/m^2^ |
| **Blood** **lipid** **score** | Non-HDL cholesterol was calculated by total cholesterol minus HDL cholesterol. Serum cholesterol was measured enzymatically. | 100 | < 130 mg/dL |
|  |  | 60 | 130-159 mg/dL |
|  |  | 40 | 160-189 mg/dL |
|  |  | 20 | 190-219 mg/dL |
|  |  | 0 | ≥ 220 mg/dL |
|  |  | If drug-treated level, subtract 20 points | |
| **Blood glucose score** | HbA1c was measured by high-performance liquid chromatography methods. | 100 | No history of diabetes |
|  |  |  | HbA1c <5.7% |
|  |  | 60 | No diabetes and HbA1c 5.7–6.4% |
|  |  | 40 | Diabetes with HbA1c <7.0% |
|  |  | 30 | Diabetes with HbA1c 7.0–7.9% |
|  |  | 20 | Diabetes with HbA1c 8.0-8.9% |
|  |  | 10 | Diabetes with HbA1c 9.0-9.9% |
|  |  | 0 | Diabetes with HbA1c ≥10.0% |
| **Blood pressure score** | The average of all available blood pressure measurements was used to calculate systolic and diastolic blood pressure. | 100 | <120/<80 mmHg |
|  |  | 75 | 120-129/<80 mmHg |
|  |  | 50 | 130-139 or 80-89 mmHg |
|  |  | 25 | 140-159 or 90-99 mmHg |
|  |  | 0 | ≥ 160 or ≥ 100 mmHg |
|  |  | Subtract 20 points (unless score is 0) if treated level | |

Abbreviation: DASH: Dietary Approaches to Stop Hypertension; HDL: High-Density Lipoprotein

## Supplemental Table 2 Definition and assessment of variables

| **Variables** | **Definition** | **Assessment** | **UK Biobank Data-Field ID** |
| --- | --- | --- | --- |
| Age (years) | Age in years. | Interval between the date of baseline assessment and the date of birth recorded by NHS. | 21003 |
| Sex | Men, Women. | NHS derived and/or touchscreen questionnaire. | 31 |
| Ethnicity | White, Others  (Mixed, Asian, Black,  Chinese, Other). | Touchscreen questionnaire: “What is your ethnic group?” | 21000 |
| Townsend deprivation index | Townsend deprivation index calculated immediately prior to participant joining UK Biobank. | Townsend Deprivation Index is a composite score of four aspects (unemployment, non-car ownership, non-home ownership, and household overcrowding), which was obtained from the residents’ postcodes using census information. | 189 |
| Education | Degree or above  (college or university  degree, other  professional  qualifications), Any other qualification, No qualification. | Touchscreen questionnaire: “Which of the following qualifications do you have?” | 6138 |
| Annual household income | Less than £31,000, Greater than or equal to £31,000, Unknown. | Touchscreen questionnaire: “What is the average total income before tax received by your HOUSEHOLD?” | 738 |
| Number of morbidities | Zero, One, Two or more. | Verbal interview of self-reported non-cancer illness. | 135 |
| Drinking status | Never, Former, Current. | Touchscreen questionnaire: “About how often do you drink alcohol?” “Did you previously drink alcohol?” | 20117 |
| Chronic kidney disease | Yes, No. | The first occurrence of any code mapped to 3-character ICD10.  N18, N18.1, N18.2, N18.3, N18.4, N18.5, and N18.9 | 132032 |
| Cardiovascular disease | Yes (including one of the following: heart failure, atrial fibrillation, coronary heart disease, stroke), No. | The first occurrence of any code mapped to 3-character ICD10.  Heart failure: I50 Atrial fibrillation: I48  Stroke: I60-I69  Coronary heart disease: I20-I25 | 131354  131350  42006  131296, 131298, 131300,131302, 131304, 131306 |

## Supplemental Table 3 The risk of chronic cardiovascular-kidney disorder according to cardiovascular health score

|  | |  | |  | | **CVH score, HR (95% CI)** | |  | | ***P* for trend** ^§^ | **PAR%** |
| --- | --- | --- | --- | --- | --- | --- | --- | --- | --- | --- | --- |
|  | |  | | **<50** | | **50-79** | | **≥80** | |  |  |
| **Total** | |  | |  | |  |  |  |  |  |  |
| **Cases/participants** | |  | | 224/10,577 | | 781/96,337 |  | 49/19,072 |  |  |  |
| **Model 1 ^*^** | |  | | 1.00 | | 0.38 (0.33-0.44) | <0.001 | 0.18 (0.13-0.25) | <0.001 |  |  |
| **Model 2** ^†^ | | | | 1.00 | | 0.42 (0.36-0.48) | <0.001 | 0.21 (0.15-0.29) | <0.001 |  |  |
| **Model 3** ^‡^ | |  | | 1.00 | | 0.46 (0.40-0.54) | <0.001 | 0.25 (0.18-0.34) | <0.001 | <0.001 | 47.4 (31.6-59.8) |
| **Diet** | |  | |  | |  |  |  |  |  |  |
| **Cases/participants** | |  | | 554/63,414 | | 241/31,559 |  | 259/31,013 |  |  |  |
| **Model 1 ^*^** | |  | | 1.00 | | 0.74 (0.64-0.86) | <0.001 | 0.72 (0.62-0.84) | <0.001 |  |  |
| **Model 2** ^†^ | | | | 1.00 | | 0.77 (0.66-0.90) | <0.001 | 0.76 (0.66-0.89) | <0.001 |  |  |
| **Model 3** ^‡^ | |  | | 1.00 | | 0.78 (0.67-0.90) | 0.001 | 0.76 (0.65-0.88) | <0.001 | <0.001 | 13.4 (3.3-22.6) |
| **Physical activity** | | |  | |  |  |  |  |  |  |  |
| **Cases/participants** | | |  | | 253/24,785 | 28/3,845 |  | 773/97,356 |  |  |  |
| **Model 1 ^*^** | | |  | | 1.00 | 0.68 (0.46-1.01) | 0.054 | 0.69 (0.60-0.80) | <0.001 |  |  |
| **Model 2** ^†^ | | | | 1.00 | | 0.72 (0.48-1.06) | 0.093 | 0.70 (0.61-0.81) | <0.001 |  |  |
| **Model 3** ^‡^ | | |  | | 1.00 | 0.74 (0.50-1.09) | 0.131 | 0.74 (0.64-0.85) | <0.001 | <0.001 | 6.5 (3.1-10.2) |
| **Nicotine exposure** | | |  | |  |  |  |  |  |  |  |
| **Cases/participants** | | |  | | 466/39,761 | 255/35,568 |  | 333/50,657 |  |  |  |
| **Model 1 ^*^** | | |  | | 1.00 | 0.69 (0.59-0.80) | <0.001 | 0.69 (0.60-0.80) | <0.001 |  |  |
| **Model 2** ^†^ | | | | 1.00 | | 0.75 (0.65-0.88) | <0.001 | 0.74 (0.64-0.85) | <0.001 |  |  |
| **Model 3** ^‡^ | | |  | | 1.00 | 0.79 (0.67-0.92) | 0.002 | 0.76 (0.66-0.88) | <0.001 | <0.001 | 10.5 (2.7-17.9) |
| **Sleep health** | | |  | |  |  |  |  |  |  |  |
| **Cases/participants** | | |  | | 90/6,177 | 183/23,035 |  | 781/96,774 |  |  |  |
| **Model 1 ^*^** | | |  | | 1.00 | 0.57 (0.44-0.74) | <0.001 | 0.53 (0.43-0.66) | <0.001 |  |  |
| **Model 2** ^†^ | | | | 1.00 | | 0.63 (0.49-0.81) | <0.001 | 0.60 (0.48-0.75) | <0.001 |  |  |
| **Model 3** ^‡^ | | |  | | 1.00 | 0.67 (0.52-0.87) | 0.002 | 0.66 (0.53-0.82) | <0.001 | 0.004 | 3.2 (-0.2-6.7) |
| **Body mass index** | | |  | |  |  |  |  |  |  |  |
| **Cases/participants** | | |  | | 374/24,026 | 466/52,570 |  | 214/49,390 |  |  |  |
| **Model 1 ^*^** | | |  | | 1.00 | 0.49 (0.43-0.57) | <0.001 | 0.29 (0.25-0.35) | <0.001 |  |  |
| **Model 2** ^†^ | | | | 1.00 | | 0.52 (0.45-0.60) | <0.001 | 0.32 (0.27-0.38) | <0.001 |  |  |
| **Model 3** ^‡^ | | |  | | 1.00 | 0.57 (0.50-0.66) | <0.001 | 0.38 (0.32-0.45) | <0.001 | <0.001 | 34.0 (26.2-41.2) |
| **Blood lipids** | | | | | |  |  |  |  |  |  |
| **Cases/participants** | 584/68,987 | | | | | 198/32,644 |  | 272/24,355 |  |  |  |
| **Model 1 ^*^** | | |  | | 1.00 | 0.89 (0.76-1.04) | 0.149 | 1.50 (1.30-1.73) | <0.001 |  |  |
| **Model 2** ^†^ | | | | 1.00 | | 0.89 (0.76-1.05) | 0.175 | 1.47 (1.27-1.70) | <0.001 |  |  |
| **Model 3** ^‡^ | | |  | | 1.00 | 0.90 (0.77-1.06) | 0.202 | 1.27 (1.09-1.46) | 0.002 | <0.001 | NA ^\|\|^ |
| **Blood glucose** | | |  | |  |  |  |  |  |  |  |
| **Cases/participants** | | |  | | 164/4,264 | 187/13,871 |  | 703/107,851 |  |  |  |
| **Model 1 ^*^** | | |  | | 1.00 | 0.36 (0.29-0.44) | <0.001 | 0.24 (0.20-0.28) | <0.001 |  |  |
| **Model 2** ^†^ | | | | 1.00 | | 0.38 (0.30-0.46) | <0.001 | 0.26 (0.22-0.31) | <0.001 |  |  |
| **Model 3** ^‡^ | | |  | | 1.00 | 0.47 (0.38-0.59) | <0.001 | 0.34 (0.29-0.41) | <0.001 | <0.001 | 10.5 (7.9-13.4) |
| **Blood pressure** | | |  | |  |  |  |  |  |  |  |
| **Cases/participants** | | |  | | 742/55,042 | 254/49,747 |  | 58/21,197 |  |  |  |
| **Model 1 ^*^** | | |  | | 1.00 | 0.58 (0.50-0.67) | <0.001 | 0.47 (0.36-0.62) | <0.001 |  |  |
| **Model 2** ^†^ | | | | 1.00 | | 0.60 (0.52-0.69) | <0.001 | 0.49 (0.37-0.64) | <0.001 |  |  |
| **Model 3** ^‡^ | | |  | | 1.00 | 0.65 (0.56-0.76) | <0.001 | 0.54 (0.41-0.71) | <0.001 | <0.001 | 32.8 (15.0-47.2) |

^*^ Adjustment for age and sex.

^†^ Adjustment for age, sex, ethnicity, Deprivation Index, education level, and annual household income.

^‡^Adjustment for age, sex, ethnicity, Deprivation Index, education level, annual household income, number of morbidities, and drinking status.

^§^ Tests for linear trends were performed by entering the median value of each category as a continuous variable in the models.

^||^ PAR% was not calculated because the adjusted HR was ≥ 1.00.

Abbreviation: CI: confidence interval; CVH: cardiovascular health; HR: hazard ratio; PAR, population attributable risk

## Supplemental Table 4 Association between CVH subscales and risk of CCV-KD

|  | **Model 1^a^** | |  | **Model 2^b^** | |  | **Model 3^c^** | |
| --- | --- | --- | --- | --- | --- | --- | --- | --- |
|  | **HR (95% CI)** | ***P*** |  | **HR (95% CI)** | ***P*** |  | **HR (95% CI)** | ***P*** |
| **Biological scale** |  |  |  |  |  |  |  |  |
| **Q1** | 1.00 |  |  | 1.00 |  |  | 1.00 |  |
| **Q2** | 0.49 (0.42-0.57) | <0.001 |  | 0.51 (0.43-0.59) | <0.001 |  | 0.54 (0.46-0.63) | <0.001 |
| **Q3** | 0.44 (0.38-0.52) | <0.001 |  | 0.47 (0.40-0.55) | <0.001 |  | 0.52 (0.45-0.61) | <0.001 |
| **Q4** | 0.30 (0.24-0.39) | <0.001 |  | 0.33 (0.26-0.42) | <0.001 |  | 0.38 (0.29-0.49) | <0.001 |
| **Behavior scale** |  |  |  |  |  |  |  |  |
| **Q1** | 1.00 |  |  | 1.00 |  |  | 1.00 |  |
| **Q2** | 0.67 (0.57-0.78) | <0.001 |  | 0.70 (0.60-0.82) | <0.001 |  | 0.73 (0.63-0.86) | <0.001 |
| **Q3** | 0.58 (0.49-0.70) | <0.001 |  | 0.63 (0.53-0.75) | <0.001 |  | 0.66 (0.55-0.79) | <0.001 |
| **Q4** | 0.49 (0.41-0.58) | <0.001 |  | 0.54 (0.45-0.65) | <0.001 |  | 0.58 (0.49-0.70) | <0.001 |

^a^ Model 1 was adjusted for age and sex. ^b^ Model 2 was adjusted for age, sex, ethnicity, deprivation Index, education level, and annual household income.

^c^ Model 3 was adjusted for age, sex, ethnicity, deprivation Index, education level, annual household income, number of morbidities, and drinking status.

Abbreviation: CI: confidence interval; CVH: cardiovascular health; CCV-KD: chronic cardiovascular-kidney disorder; HR: hazard ratio

## Supplemental Table 5 Association between CVH quartiles and risk of CCV-KD

|  | **Model 1^a^** | |  | **Model 2^b^** | |  | **Model 3^c^** | |
| --- | --- | --- | --- | --- | --- | --- | --- | --- |
|  | **HR (95% CI)** | ***P*** |  | **HR (95% CI)** | ***P*** |  | **HR (95% CI)** | ***P*** |
| **Q1** | 1.00 |  |  | 1.00 |  |  | 1.00 |  |
| **Q2** | 0.57 (0.49-0.66) | <0.001 |  | 0.60 (0.52-0.69) | <0.001 |  | 0.63 (0.55-0.74) | <0.001 |
| **Q3** | 0.45 (0.38-0.53) | <0.001 |  | 0.48 (0.41-0.57) | <0.001 |  | 0.53 (0.44-0.62) | <0.001 |
| **Q4** | 0.26 (0.21-0.32) | <0.001 |  | 0.29 (0.23-0.36) | <0.001 |  | 0.33 (0.26-0.41) | <0.001 |
| **HR per 10-point increase** | 0.65 (0.62-0.68) | <0.001 |  | 0.67 (0.64-0.71) | <0.001 |  | 0.70 (0.67-0.74) | <0.001 |

^a^ Model 1 was adjusted for age and sex. ^b^ Model 2 was adjusted for age, sex, ethnicity, deprivation Index, education level, and annual household income.

^c^ Model 3 was adjusted for age, sex, ethnicity, deprivation Index, education level, annual household income, number of morbidities, and drinking status.

Abbreviation: CI: confidence interval; CVH: cardiovascular health; CCV-KD: chronic cardiovascular-kidney disorder; HR: hazard ratio

## Supplemental Table 6 Association between CVH categories and risk of CCV-KD in subgroups

|  | **Low CVH** | **Intermediate CVH** | **High CVH** | ***P*** |
| --- | --- | --- | --- | --- |
| **Age (y)** |  |  |  | 0.610 |
| **<60** | 1.00 | 0.52 (0.38-0.72) | 0.22 (0.12-0.42) |  |
| **≥60** | 1.00 | 0.45 (0.38-0.54) | 0.25 (0.17-0.36) |  |
| **Sex** |  |  |  | 0.255 |
| **Male** | 1.00 | 0.49 (0.41-0.59) | 0.30 (0.20-0.46) |  |
| **Female** | 1.00 | 0.42 (0.32-0.55) | 0.18 (0.11-0.29) |  |
| **Ethnicity** |  |  |  | 0.915 |
| **White** | 1.00 | 0.46 (0.40-0.54) | 0.24 (0.17-0.33) |  |
| **Others** | 1.00 | 0.64 (0.27-1.51) | 0.25 (0.03-2.07) |  |
| **Deprivation** |  |  |  | 0.558 |
| **<median** | 1.00 | 0.50 (0.40-0.63) | 0.28 (0.18-0.43) |  |
| **≥median** | 1.00 | 0.44 (0.36-0.54) | 0.20 (0.12-0.32) |  |
| **Education level** |  |  |  | 0.539 |
| **University or college degree** | 1.00 | 0.43 (0.33-0.56) | 0.25 (0.16-0.39) |  |
| **Others** | 1.00 | 0.49 (0.41-0.59) | 0.22 (0.14-0.34) |  |
| **Annual household income (£)** | | | | 0.708 |
| **<31,000** | 1.00 | 0.45 (0.37-0.55) | 0.18 (0.11-0.29) |  |
| **≥31,000** | 1.00 | 0.47 (0.37-0.61) | 0.28 (0.17-0.45) |  |
| **Unknown** | 1.00 | 0.58 (0.33-1.00) | 0.43 (0.17-1.07) |  |
| **Multimorbidity** |  |  |  | 0.832 |
| **0** | 1.00 | 0.39 (0.22-0.66) | 0.14 (0.06-0.37) |  |
| **1** | 1.00 | 0.46 (0.31-0.68) | 0.27 (0.14-0.52) |  |
| **≥2** | 1.00 | 0.48 (0.40-0.57) | 0.25 (0.17-0.37) |  |
| **Drinking status** |  |  |  | 0.754 |
| **Current** | 1.00 | 0.46 (0.40-0.54) | 0.24 (0.18-0.34) |  |
| **Former** | 1.00 | 0.78 (0.35-1.72) | 0.24 (0.03-1.96) |  |
| **Never** | 1.00 | 0.36 (0.17-0.76) | 0.11 (0.02-0.54) |  |

Abbreviation: CVH: cardiovascular health; CCV-KD: chronic cardiovascular-kidney disorder

## Supplemental Table 7 Association between CVH categories and risk of CCV-KD using competing risk models

|  | **Model 1^a^** |  | **Model 2^b^** | | | **Model 3^c^** | |
| --- | --- | --- | --- | --- | --- | --- | --- |
|  | **HR（95% CI）** | ***P*** | **HR（95% CI）** | ***P*** | **HR（95% CI）** | | ***P*** |
| **Low CVH** | 1.00 |  | 1.00 |  | 1.00 | |  |
| **Intermediate CVH** | 0.39 (0.34-0.44) | <0.001 | 0.43 (0.36-0.48) | <0.001 | 0.47 (0.41-0.54) | | <0.001 |
| **High CVH** | 0.19 (0.14-0.25) | <0.001 | 0.22 (0.16-0.29) | <0.001 | 0.26 (0.20-0.35) | | <0.001 |

^a^ Model 1 was adjusted for age and sex. ^b^ Model 2 was adjusted for age, sex, ethnicity, deprivation Index, education level, and annual household income.

^c^ Model 3 was adjusted for age, sex, ethnicity, deprivation Index, education level, annual household income, number of morbidities, and drinking status.

Abbreviation: CI: confidence interval; CVH: cardiovascular health; CCV-KD: chronic cardiovascular-kidney disorder; HR: hazard ratio

## Supplemental Table 8 Association between CVH categories and risk of CCV-KD excluding events within the first two years of follow-up

|  | **Model 1^a^** |  | **Model 2^b^** | | **Model 3^c^** | |
| --- | --- | --- | --- | --- | --- | --- |
|  | **HR（95% CI）** | ***P*** | **HR（95% CI）** | ***P*** | **HR（95% CI）** | ***P*** |
| **Low CVH** | 1.00 |  | 1.00 |  | 1.00 |  |
| **Intermediate CVH** | 0.38 (0.33-0.45) | <0.001 | 0.42 (0.36-0.48) | <0.001 | 0.46 (0.40-0.54) | <0.001 |
| **High CVH** | 0.18 (0.13-0.25) | <0.001 | 0.21 (0.15-0.29) | <0.001 | 0.25 (0.18-0.35) | <0.001 |

^a^ Model 1 was adjusted for age and sex. ^b^ Model 2 was adjusted for age, sex, ethnicity, deprivation Index, education level, and annual household income.

^c^ Model 3 was adjusted for age, sex, ethnicity, deprivation Index, education level, annual household income, number of morbidities, and drinking status.

Abbreviation: CI: confidence interval; CVH: cardiovascular health; CCV-KD: chronic cardiovascular-kidney disorder; HR: hazard ratio

## Supplemental Table 9 Association between CVH categories and risk of CCV-KD after imputing missing values on covariates

|  | **Model 1^a^** |  | **Model 2^b^** | | **Model 3^c^** | |
| --- | --- | --- | --- | --- | --- | --- |
|  | **HR（95% CI）** | ***P*** | **HR（95% CI）** | ***P*** | **HR（95% CI）** | ***P*** |
| **Low CVH** | 1.00 |  | 1.00 |  | 1.00 |  |
| **Intermediate CVH** | 0.38 (0.33-0.44) | <0.001 | 0.41 (0.36-0.47) | <0.001 | 0.46 (0.40-0.53) | <0.001 |
| **High CVH** | 0.18 (0.14-0.24) | <0.001 | 0.21 (0.16-0.28) | <0.001 | 0.25 (0.19-0.34) | <0.001 |

^a^ Model 1 was adjusted for age and sex. ^b^ Model 2 was adjusted for age, sex, ethnicity, deprivation Index, education level, and annual household income.

^c^ Model 3 was adjusted for age, sex, ethnicity, deprivation Index, education level, annual household income, number of morbidities, and drinking status.

Abbreviation: CI: confidence interval; CVH: cardiovascular health; CCV-KD: chronic cardiovascular-kidney disorder; HR: hazard ratio

## Supplemental Table 10 The risk of chronic cardiovascular-kidney disorder separated by the disease diagnosed first according to CVH score

|  |  | |  | **CVH score, HR (95% CI)** | |  | |
| --- | --- | --- | --- | --- | --- | --- | --- |
|  |  | | **<50** | **50-79** | | **≥80** | |
| **Overall** |  | |  |  | |  | |
| **CKD first** | | | 1.00 | 0.44 (0.33, 0.58) | <0.001 | 0.11 (0.05, 0.25) | <0.001 |
| **CVD first** |  | | 1.00 | 0.41 (0.34, 0.51) | <0.001 | 0.23 (0.15, 0.35) | <0.001 |
| **Diet** |  | |  |  |  |  |  |
| **CKD first** | | | 1.00 | 0.62 (0.46, 0.83) | 0.001 | 0.60 (0.45, 0.80) | 0.001 |
| **CVD first** |  | | 1.00 | 0.74 (0.60, 0.91) | 0.005 | 0.79 (0.65, 0.97) | 0.022 |
| **Physical activity** | |  |  |  |  |  |  |
| **CKD first** | | | 1.00 | 0.42 (0.17, 1.04) | 0.060 | 0.63 (0.49, 0.81) | <0.001 |
| **CVD first** | |  | 1.00 | 0.84 (0.51, 1.39) | 0.502 | 0.75 (0.61, 0.91) | 0.003 |
| **Nicotine exposure** | |  |  |  |  |  |  |
| **CKD first** | | | 1.00 | 0.75 (0.56, 1.00) | 0.051 | 0.66 (0.50, 0.87) | 0.003 |
| **CVD first** | |  | 1.00 | 0.77 (0.63, 0.95) | 0.016 | 0.74 (0.61, 0.90) | 0.003 |
| **Sleep health** | |  |  |  |  |  |  |
| **CKD first** | | | 1.00 | 0.99 (0.61, 1.61) | 0.963 | 0.77 (0.49, 1.20) | 0.243 |
| **CVD first** | |  | 1.00 | 0.53 (0.38, 0.74) | <0.001 | 0.58 (0.44, 0.77) | <0.001 |
| **Body mass index** | |  |  |  |  |  |  |
| **CKD first** | | | 1.00 | 0.47 (0.36, 0.61) | <0.001 | 0.37 (0.27, 0.51) | <0.001 |
| **CVD first** | |  | 1.00 | 0.68 (0.56, 0.82) | <0.001 | 0.37 (0.29, 0.47) | <0.001 |
| **Blood lipids** | | | |  |  |  |  |
| **CKD first** | | | 1.00 | 0.85 (0.62, 1.16) | 0.295 | 1.46 (1.12, 1.91) | 0.005 |
| **CVD first** | |  | 1.00 | 0.86 (0.68, 1.07) | 0.168 | 1.16 (0.95, 1.42) | 0.156 |
| **Blood glucose** | |  |  |  |  |  |  |
| **CKD first** | | | 1.00 | 0.47 (0.33, 0.68) | <0.001 | 0.25 (0.18, 0.35) | <0.001 |
| **CVD first** | |  | 1.00 | 0.51 (0.38, 0.69) | <0.001 | 0.39 (0.30, 0.50) | <0.001 |
| **Blood pressure** | |  |  |  |  |  |  |
| **CKD first** | | | 1.00 | 0.68 (0.52, 0.89) | 0.005 | 0.57 (0.35, 0.93) | 0.025 |
| **CVD first** | |  | 1.00 | 0.61 (0.50, 0.75) | <0.001 | 0.51 (0.34, 0.74) | 0.001 |

Note: Adjustment for age, sex, ethnicity, Deprivation Index, education level, annual household income, number of morbidities, and drinking status.

## Supplemental Figure 1 Flowchart of the study


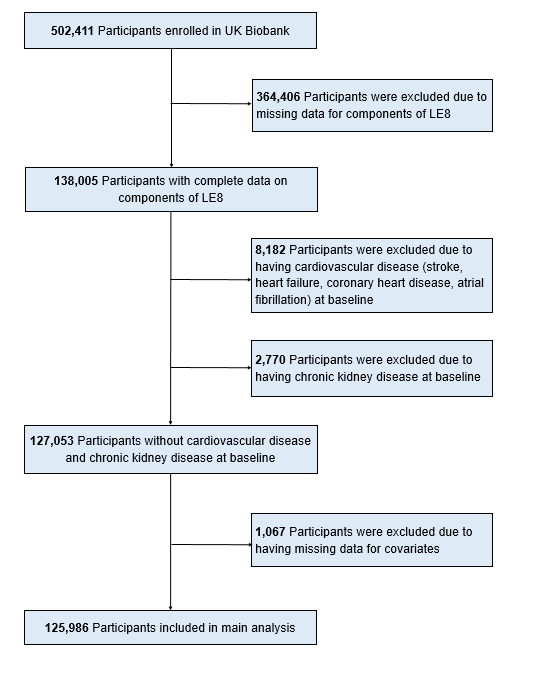


## Supplemental Figure 2 Kaplan-Meier curve for cumulative incidence of chronic cardiovascular-kidney disorder by cardiovascular health category


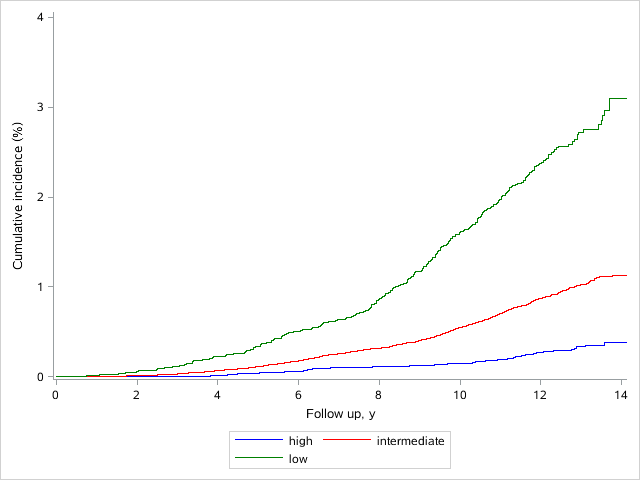

Supplement: Supplementary file 1 — Supplementary Material 1. [file 12889_2024_19532_MOESM1_ESM.docx]
